# Assessing the effect of environmental and socio-economic factors on skin melanoma incidence: an island-wide spatial study in Gran Canaria (Spain), 2007–2018

**DOI:** 10.1007/s10552-022-01614-6

**Published:** 2022-08-04

**Authors:** Mercè Grau-Pérez, Leopoldo Borrego, Gregorio Carretero, Pablo Almeida, Jorge Cano

**Affiliations:** 1 Universidad de Las Palmas de Gran Canaria (ULPGC), Calle Juan de Quesada 30, 35001 Las Palmas de Gran Canaria, Spain; 2 Dermatology Department, Hospital Universitario Puerta de Hierro, Majadahonda, Spain; 3 Dermatology Department, Hospital Universitario de Gran Canaria Doctor Negrín, Las Palmas de Gran Canaria, Spain; 4 Dermatology Department, Complejo Hospitalario Universitario Insular-Materno Infantil de Gran Canaria, Las Palmas de Gran Canaria, Spain; 5 Expanded Special Project for Elimination of Neglected Tropical Diseases (ESPEN), World Health Organization’s Regional Office for Africa, Brazzaville, Republic of the Congo

**Keywords:** Melanoma, Epidemiology, Spatial analysis, Socio-economic factors, Environment, Spain

## Abstract

**Introduction:**

Skin melanoma incidence has risen in the last decades becoming a major public health problem in many regions of the world. Geographic variation of rates is not well understood.

**Purpose:**

To assess the spatial distribution of skin melanoma in Gran Canaria Island (Canary Islands, Spain) and to evaluate the role of environmental, socio-economic, and demographic factors in this distribution.

**Methods:**

We performed a small-area study with disease mapping at the census-tract level (CT) in Gran Canaria between 2007 and 2018. After testing for spatial autocorrelation, we integrated individual-level health data with census-based demographic and socio-economic indicators, and satellite-based environmental data. Finally, we assessed the role of demographic, socio-economic and environmental factors on skin melanoma incidence using a Bayesian analytical framework, with options for non-spatial and spatial random effects.

**Results:**

1058 patients were diagnosed with invasive skin melanoma in the study period and geolocated to a CT (number of CT in Gran Canaria = 565). We found evidence of global spatial autocorrelation in skin melanoma incidence (Moran’s *I* = 0.09, pseudo *p*-value = 0.001). A few hotspots were detected, fundamentally in urban northern tracts. A radial pattern of high values was also observed in selected ravines with historical isolation. Multivariable conditional autoregressive models identified urbanicity, percent of females, and a high socio-economic status as risk factors for disease. Solar radiation did not show a significant role.

**Conclusion:**

Urbanicity and a high socio-economic status were identified as the main risk factors for skin melanoma. These associations might reflect differential melanoma susceptibilities or be explained by health inequalities in detection. This study also uncovered high-risk areas in particular ravines. Future targeted research in these regions might help better understand the role of genetic and toxic factors in melanoma pathogenesis.

**Supplementary Information:**

The online version contains supplementary material available at 10.1007/s10552-022-01614-6.

## Introduction

Geographic variability in skin melanoma incidence is a long-standing observation, with the highest rates occurring in Australasia, North-western Europe, and Northern America [1]. Environmental, phenotypic, and socio-economic factors are thought to explain this variation [2, 3] that is still poorly understood.

Excessive ultraviolet radiation (UV) exposure among fair-skinned populations has been identified as the main environmental risk factor to develop disease [4] and high socio-economic status (SES) populations have consistently been associated with increased incidence rates [3], which has given rise to the concepts of a latitudinal and a social gradient in melanoma distribution [5–7]. However, many regions escape these gradients and, despite the potential confounding role that environmental and socio-demographic factors could have for one another, the influence of both types of risk factors has deserved little attention [8, 9].

The evolution of spatial epidemiology in the last two decades as Geographic Information Systems (GIS) have become more available, together with the advent of methodological and computational developments, has enabled the assessment of geographic variations of disease with a new scope [10]. By allowing to integrate and link data from multiple sources at a small-area level, spatial methods have become an asset in cancer surveillance and as a hypothesis generating tool [11].

The Canary Islands are a Spanish archipelago located in the Atlantic Ocean off the coast of North-western Africa, with a subtropical climate. The population in the islands is exposed to a high environmental risk for the development of skin cancers [12], yet unexpectedly the incidence of skin melanoma in the region is among the lowest in Spain and below the estimates provided by the World Health Organization for Southern Europe [1, 13].

Mapping skin melanoma distribution in Gran Canaria, one of the main Islands, and evaluating the factors driving this geographic distribution could provide insight into this unexpected epidemiological finding and contribute to disentangle the role of environmental and socio-economic factors in melanoma incidence at a small geographic scale.

In this study, we assessed the spatial heterogeneity of skin melanoma incidence at the census-tract (CT) level across Gran Canaria in the period 2007–2018. In addition, we aimed at identifying the occurrence of potential hotspots, namely areas at higher risk of skin melanoma across the island, and at evaluating whether demographic, socio-economic, and environmental variables could represent risk factors for skin melanoma occurrence and explain its spatial variation.

## Methods

### Study site and study population

Gran Canaria is one of the two main islands of the Canary Islands archipelago. With a population of about 850,000 inhabitants, its area is relatively small (1,560 km^2^), yet the altitude range is high (0–1,956 m), reflecting its volcanic origin. With an almost circular shape of diameter 47 km, it can be topographically divided into ravines that start in the central summit down to the coast, in a radial configuration (Supplementary Fig. S1) [14]. The capital city of Las Palmas de Gran Canaria (LPGC) is located in the northeast corner of the island and concentrates about half of the island’s population.

Universal health coverage is available in the region and delivered through the government-funded Canary Islands Healthcare Service (CIHS). Access to dermatologic care in the CIHS is available through a General Practitioner (GP) referral, and GPs are assigned based on residential address.

### Data sources

Skin melanoma cases were obtained from the Gran Canaria skin melanoma dataset specified below, and covariate data were downloaded from the open-data sources listed in supplementary Table S1. A detailed description of the datasets is also provided.

#### Skin melanoma cases

The Gran Canaria skin melanoma dataset used in this study has been previously described in detail [13]. It includes demographic, clinical, and histological information of all invasive skin melanoma cases evaluated at the CIHS among GC residents, diagnosed between January 2007 and December 2018. It has a comprehensive geographic coverage within the island and contains all cases diagnosed in the public CIHS facilities (which represent 86% of all healthcare in the region) [15] and most cases diagnosed in the private setting. It was compiled following the International Classification of Diseases ICD-10 diagnosis code C43 (malignant melanoma of skin), thereby excluding in situ and mucosal melanomas. In case multiple melanomas were diagnosed in a patient, only the first one was counted, in accordance with the International Agency for Research on Cancer rules [16].

The information about the residential address of patients at the time of diagnosis was retrieved from the clinical records before the dataset was de-identified for further analyses. Cases were located at the street level and then reassigned to a CT by georeferencing the street.

#### Spatial unit

CTs are the smallest geographic unit for which census data are available in Spain. Population data for Gran Canaria and the outline of CTs were downloaded from the Spanish National Institute of Statistics (INE) and corresponded to the Spanish Population and Housing Censuses of 2011 (SPHC2011)[17].

In Spain, the census is updated every ten years. SPHC2011 was the first census based on a sampling survey and included 12.3% of the population. Surface areas of CTs in Gran Canaria varied from 8,798 to 12,306,130 m^2^, the population size range was 320 to 5,565 inhabitants and the number of CTs 565.

#### Demographic and socio-economic variables

Demographic (age and sex composition of the population) and educational attainment indicators were obtained from the SPHC2011 database.

Some additional economic indicators, such as unemployment rates, income type, and rent, were obtained from  Spain’s Household income distribution atlas for 2015 at the same geographic scale (CT level) [18].

In addition, we also considered the first deprivation index with full geographic coverage in Spain (IP2011) that has recently been made publicly available [19]. Based on socio-economic indicators from the SPHC2011 and elaborated using principal component analysis, IP2011 integrates information regarding percentages of the following indicators: manual workers, temporary workers, unemployment, insufficient (incomplete compulsory) education overall, insufficient education among youth (16 to 29-year-olds), and dwellings without internet access [19]. Constructed as a quantitative variable at the CT level, it is a standardized index with a mean of 0 and standard deviation of 1 and is to be interpreted on relative terms, with values close to 0 indicating the average deprivation of the country, positive values indicating more deprivation (meaning more poverty) and negative values less deprivation.

#### Environmental variables

Cartographic data included administrative boundaries, roads, water surfaces, and altitude data. Land cover information was obtained from the European Environmental Agency’s satellite data of the Copernicus Land Monitoring Service [20]. Solar radiation data were downloaded from the global climate and weather data WorldClim (version 2.0) [21].

### Data preparation

#### Georeferencing skin melanoma cases

We georeferenced the residential addresses of cases at the street level with two widely used Application Programming interfaces (APIs): OpenCage geocoder and Google Earth Engine, extracting longitude and latitude for each occurrence with both APIs. For this, we used the packages *opencage v0.2.2* and *ggmap v3.0.0* in R v3.6.3 [22–24]. We mapped coordinates to cross-check for spatial consistency. Discordant cases and those with potential geolocation errors were manually geolocated with OpenStreetMap. As a second step, we aggregated the spatial point data to the CT level. Gran Canaria is divided into 565 CTs, yet when linking the shapefile to the census, three tracts did not exist and were merged to the corresponding ones with a final number of 562 tracts. A shapefile was created with these aggregated data.

#### Spatial covariates: potential predictors of the distribution of cases

The proportion of females per CT was obtained by dividing the total number of females by the total population, within each CT.

Regarding SES variables available at the census, we generated a new variable named *p_basic* (basic studies) by aggregating the variables: (i) illiterate persons, (ii) those without studies (less than 5 years of schooling), and (iii) those with first-level studies (elementary school completed, as higher educational attainment). For each CT, all variables of educational attainment were divided by the population aged over 16 years, to obtain a proportion of the population with different degrees of educational attainment within each CT (*p_basic*, *p_2_grd*, and *p_3_grd*, supplementary Tables S1, S2). Economic indicators of 2015 were aggregated to their corresponding CT.

The deprivation index covariate had the same geographic coverage as the other SES variables and was linked to the census shapefile without incidences.

Land cover and climatic data were downloaded at a global scale in a raster format (continuous surface divided into regular grids) and extracted for the Canary Islands with the Mask feature of ArcGIS and projected in ETRS89/UTM zone 30 N (EPSG: 25830) at 1km^2^ resolution. Land cover data originally had 44 different categories and were obtained at 100-m resolution. It was expected that data scarcity for many categories would be troublesome, therefore a simplified Corine classification with broader categories was used, those being: artificial surfaces (urban), agricultural areas, and forests and semi-natural areas. In case a CT contained many categories, it was assigned the modal value (most frequent category). In order to allow for modeling with binary or numeric variables, a further simplified categorization of these three categories was performed, finally using percent of urban land and percent of non-urban land (agricultural, forests and semi-natural areas).

Hillshade and slope rasters were created using the corresponding ArcGIS Spatial Analysis tools, by integrating the information about altitude and azimuth from cartographic data.

For rasters with numeric values (Hillshade, solar radiation, digital elevation, and slope), the mean value for each cell was extracted. Input grids were resampled to a common spatial resolution of 1 km^2^ using the nearest neighbor approach and clipped to match the geographic extent of a map of Gran Canaria and eventually aligned to it. Raster manipulation and processing were undertaken using *raster* [26] package in R and final map layouts created with ArcGIS 10.8 software [25].

Missing data for covariates were scarce (the maximum missing data of a covariate occurred in 16 CTs). For missing values, multiple imputation was performed with *mice* [27] R package, by means of a linear regression using bootstrapping.

### Statistical analyses

#### Outcome definition

The outcome of this study was the spatial distribution of skin melanoma incidence in Gran Canaria in 2007–2018, measured as standardized incidence ratios (SIR) per CT for the study period. SIR is defined as a ratio of the number of observed cases to the number that would be expected, if the study population experienced the same incidence rates as the reference population. In our study, the reference population was the total population of Gran Canaria. An SIR was calculated for each CT, taking the number of cases and the CT population into account: SIR_i_ = Observed_i_/Expected_i_, where *O*_i_ is the observed number of cases, *E*_i_ = *rP*_i_ is the expected number of cases, *P*_i_ is the population, and *r* = sum of (observed cases/total population) is the overall incidence ratio. An SIR of 1 indicated an incidence equal to that expected for the CT, based on the overall crude incidence in the island.

Skin melanoma incidence is known to increase with age. To account for the potential confounding effect that age composition per CT might have, expected counts were calculated for each age group category available at the census (< 16, 16 to 64, and > 64 years) and then added, to obtain an age-adjusted SIR (aSIR) = Observed cases/Age-adjusted expected cases, for each CT [28]. An aSIR of 1 indicates an incidence equal to that expected for the CT, taking into account the total population of the CT and its age composition. An aSIR > 1 indicates a higher incidence than expected, and an aSIR < 1 a lower incidence than expected.

#### Analysis of spatial clustering

We initially tested for global spatial autocorrelation with Moran’s *I* statistic using GeoDA software [29], to identify whether geographic variations in incidence were random or whether there was evidence for spatial clustering in the data.

The spatial weights matrix used to define the spatial relationships of the CTs was based on Queen’s contiguity, with a spatial lag of one (first-order adjacency). Queen contiguity defines neighbors as spatial units sharing a common edge or a common vertex and seemed more appropriate for Gran Canaria than the available alternatives due to the geographic variability of tracts in morphology and size. Monte Carlo simulation was performed in order to test the statistical significance of Moran’s *I* coefficient. 999 permutations were used to obtain more stable pseudo *p*-values.

In case evidence for spatial autocorrelation was found, local spatial autocorrelation with Local Moran’s *I* statistic (LISA) would be performed to check for the presence of clusters/outliers, and Monte Carlo methods applied again. We performed sensitivity analyses and increased the significance level to 0.01 to decrease the risk of false positives arising from multiple comparisons.

#### Bayesian statistical modeling

CTs are small areas, and limitations of SIR comparisons across small areas have previously been noted. Bayesian modeling methods allow to adjust for data from neighboring small areas and to obtain a quantification of the uncertainty around the estimates, improving former approaches [30]. Since the dataset was too sparse to fit a spatiotemporal model due to the relatively low melanoma incidence in the region, annual cases were aggregated to consider a spatial model for the whole study period (2007–2018).

We modeled the incidence of skin melanoma in Gran Canaria by fitting multivariable spatial generalized linear mixed models with inference in a Bayesian setting, using Integrated Nested Laplace Approximations. Models were implemented with *INLA* and *CARBayes* packages in R [22, 31, 32]. The response variable, aSIR, was fitted using multiple Bayesian Poisson models with a range of explanatory variables as potential risk factors (fixed effects) and different options for random effects with (i) no random effects (Model 1), (ii) independent random effects (Model 2), and (iii) spatially correlated random effects, implemented through a conditional autoregressive model (CAR, Model 3). Independent random effects are a tool conceived for modeling and structuring the sources of variability underlying the data [33]. Model 2, fitted with the effect of covariates and with independent random effects, provided a baseline to assess whether residual spatial dependence (added in model 3) was required to model the data [34]. Model 3 was fitted using the widely used Besag, York, and Mollié (BYM) spatial model [35, 36].

For the selection of covariates, based on previous evidence we decided a priori that an adjustment for age, sex, and SES needed to be included in the final model, apart from the effect of environmental variables that we aimed to test [3, 13]. Age was accounted for by modeling age-adjusted SIR, and sex by adjusting for percent of females per CT. For the other predictors, we explored the distribution of each covariate and fitted univariable models using simple Poisson regression without accounting for random effects. A correlogram of all covariates was performed to (i) evaluate the relationship between them, (ii) assess how the deprivation index IP2011 behaved compared to other SES measures in the region, and (iii) exclude correlated covariates in order to reduce the risk of collinearity and overfitting in the final model (see Supplementary Fig. S3). All covariates were scaled ((value-mean)/standard deviation) and then the selected ones fitted as fixed effects in all models.

The adequacy of the fitted models was explored using standard posterior predictive checks. A final comparison was made to select the best model using the deviance information criterion (DIC), i.e., the one with a lowest DIC [37]. Details on the specifications of these Bayesian Poisson models are given in the supplementary file (Supplementary Text T1).

#### Ethical clearances

The study was approved by the regional Research Ethics Committee (CEI/CEIm HUGCDN, Code 2019-515-1).

## Results

**Fig. 1:**
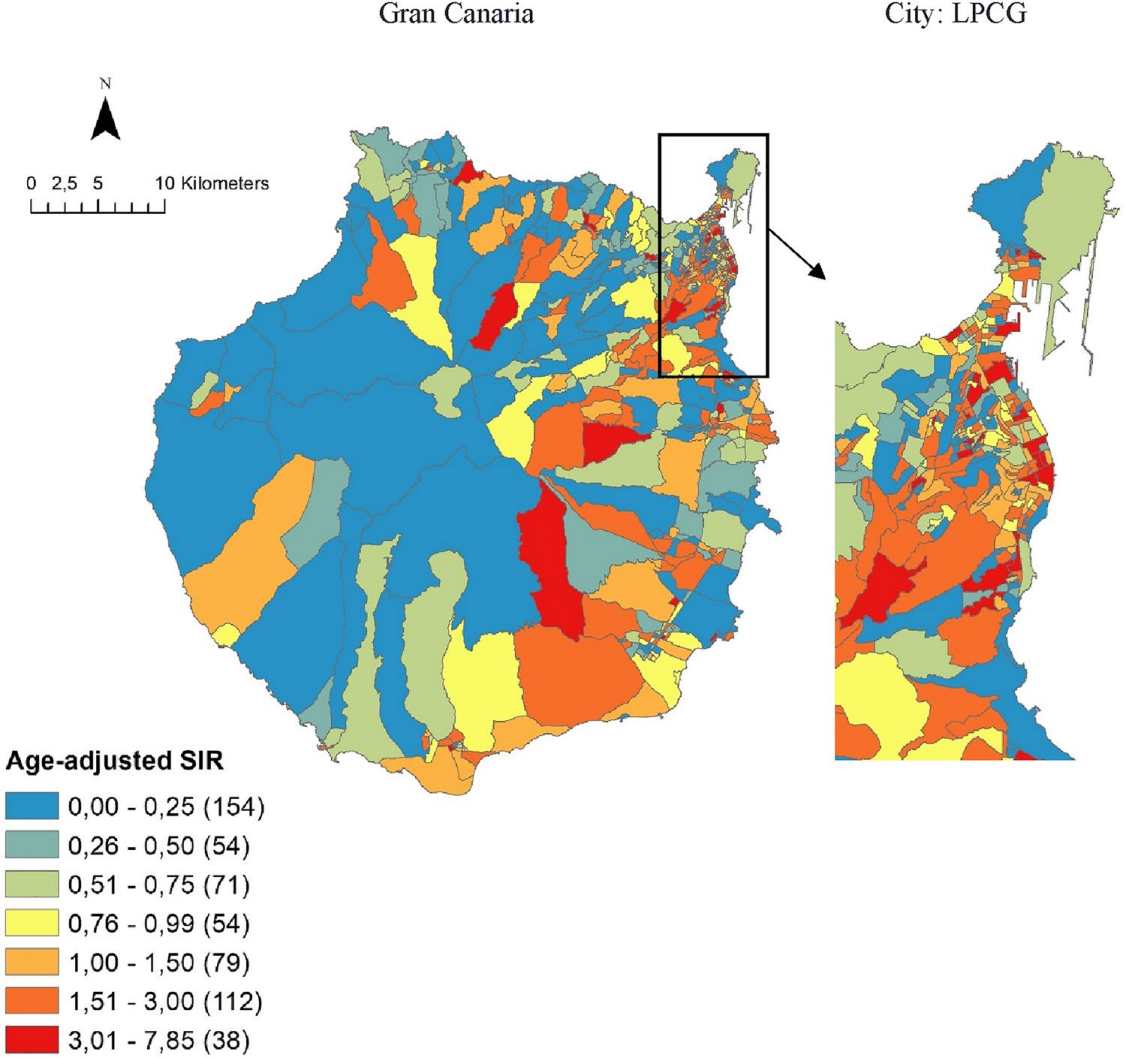
Choropleth map showing the incidence of skin melanoma in Gran Canaria Island and in the capital city of Las Palmas de Gran Canaria (LPGC) in 2007–2018, at the census-tract level. Incidence presented as Age-adjusted Standardized Incidence Ratios (aSIR) per census tract (CT). An aSIR of 1 in a CT indicates an observed number of cases equal to that expected for the CT in the study period, based on the overall incidence in the island and considering the total population of the CT and its age composition. An aSIR > 1 indicates a higher incidence than expected, and aSIR < 1 a lower incidence than expected. An aSIR of 3 indicates an incidence 3 times higher than expected for the CT

In the period 2007–2018, 1,058 skin melanoma cases were diagnosed among Gran Canaria residents, of which 1,055 (99.7%) could be ascribed to a CT and mapped. The overall incidence rate was of 10.4 cases per 100,000 person-years. The age-specific rate in the 16- to 64-year-old age stratum was of 9.4 cases per 100,000 person-years, while in people older than 64, the rate rose to 29.4. No cases were diagnosed in people younger than 16 in the whole study period. Age-adjusted SIR (aSIR) were obtained for each CT across the island, with a median of 0.77 (IQR 0.00–1.56) and a maximum value of 7.85. The distribution of aSIR across the island is presented in Fig. 1, where high values can be observed in some CTs of the capital city (LPGC) and following selected ravines of the island (Tirajana, Telde, Azuaje, and end of Guiniguada ravines, supplementary Fig. S1).

We found evidence of spatial heterogeneity in the distribution of skin melanoma incidence across the island. Moran’s *I* coefficient of spatial global autocorrelation indicated positive clustering with SIR (*I* = 0.09, pseudo p-value = 0.001), meaning cases were more spatially clustered than expected by chance, and neighboring tracts tended to have more similar incidences than distant tracts. After adjusting for the effect of age using aSIR, there was still evidence for spatial autocorrelation although the magnitude was lower (*I* = 0.06, pseudo *p*-value = 0.009).

**Fig. 2:**
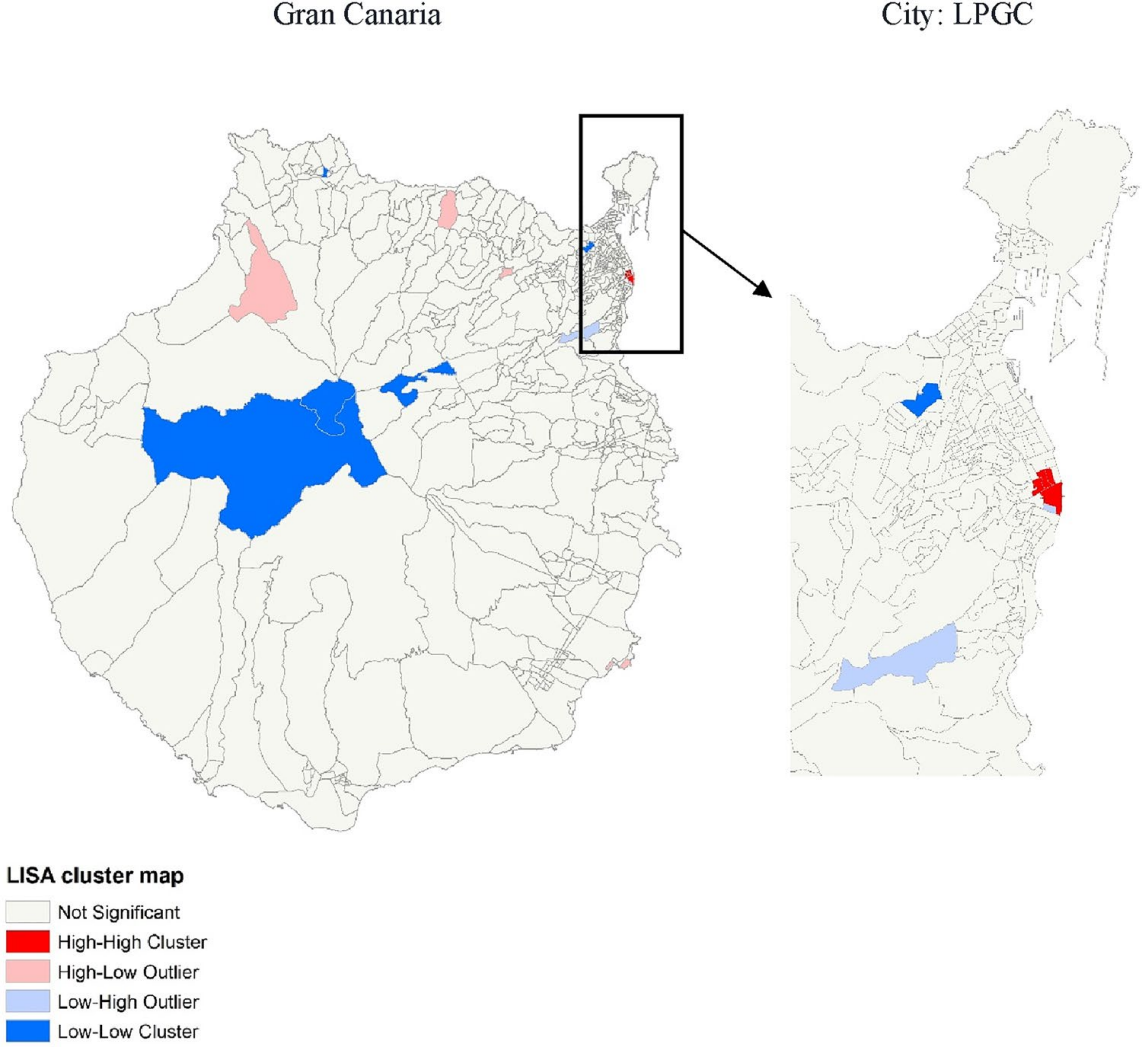
Cluster maps of skin melanoma incidence showing the type of spatial association between adjacent neighborhoods in Gran Canaria and in LPGC city in 2017–2018. Local Indicator of Spatial Autocorrelation (LISA) cluster map. High–high and low–low indicate clustering of similar values and high–low and low–high indicate spatial outliers. Spatial weights with Queen’s contiguity of first order, with age-adjusted SIR as outcome and significance filter of *p* < 0.01

Figure 2 shows the results of the analysis of local spatial autocorrelation (LISA), highlighting CTs that are clustering based on measured aSIR. A few areas at increased risk (hot spots) were identified in the northern region of the island, particularly in Triana, an urban, high-SES area of LPGC and in neighborhoods of Agaete and Firgas municipalities. Clusters of low aSIR (cold spots) were also identified in more deprived, central rural neighborhoods of Tejeda and La Vega de San Mateo and in the northern municipality of Santa Maria de Guía (Fig. 2 and supplementary Figs. S1 and S2).

Maps showing the distribution of covariates at the CT level, and correlation plots of covariates are presented in supplementary Figs. S2 and S3. Regarding SES variables, IP2011 showed correlation (Pearson’s correlation coefficient > 0.5 or < -0.5) with both economic and educational attainment variables, including those from the SPHC2011 used to elaborate the index itself and with the supplementary economic covariates of Spain’s Household income distribution atlas. Therefore, IP2011 was selected as the covariate to adjust for SES in further modeling steps.

Urbanization, measured as the percentage of CT area covered by urban land, showed strong negative correlation with altitude, slope, and other land cover types and was selected as the single land cover and topographic variable, as it was deemed as potentially more relevant based on previous findings [38, 39]. Solar radiation and Hillshade did not show collinearity with other potential explanatory factors and were then selected for further analyses.

Bayesian Poisson regression models of aSIR were fitted with the selected explanatory covariates (deprivation index, percent of females, percent of urbanized land, solar radiation) and with different options for random effects implemented through a BYM CAR model. The model accounting for independent random effects (model 2) outperformed the model with only fixed effects (Table 1). There was no evidence of spatial autocorrelation in the residuals, indicating that the covariates reasonably accounted for the existing spatial structure in the aSIR.

**Table 1:** Deviance information criteria (DIC) values for the different types of Bayesian Poisson models

Model type	DIC values
Model 1: No random effects	2088.7
Model 2: Independent random effects	1929.5
Model 3: Globally smoothed CAR (global spatial smoothing)	1928.6
Model 4: Locally smoothed CAR (local spatial smoothing)	1940.5

*CAR:* conditional autoregressive model. All models include fixed effects (i.e. the covariates: age, %females, deprivation index, % urban land, and solar radiation)

**Fig. 3:**
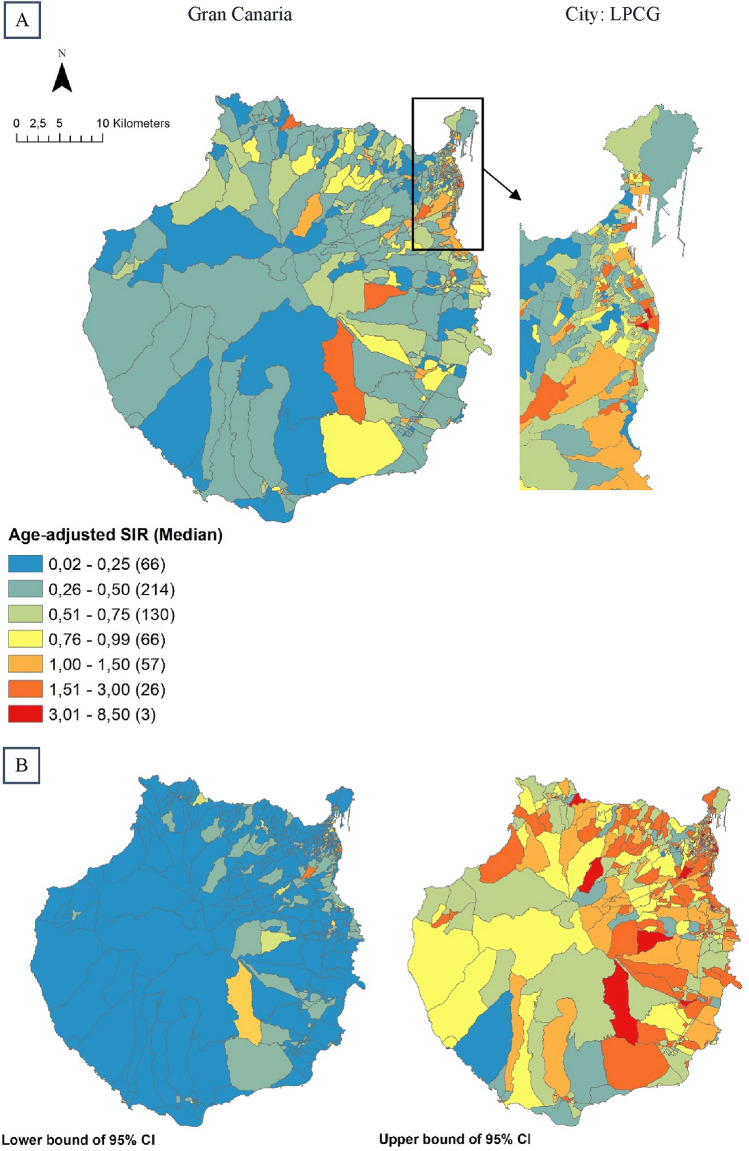
Modeled skin melanoma incidence in Gran Canaria Island and in the city of Las Palmas de Gran Canaria (LPGC) in 2007–2018 at the census-tract level, with the final model. **A** Fitted values of median aSIR with the Bayesian conditional autoregressive model accounting for fixed and independent random effects. **B** Lower and upper bounds of the 95% CI for modeled aSIR across the island. *aSIR* age-adjusted standardized incidence ratios, *CI* credible interval

Bayesian CAR models with spatially structured random effects (global and local spatial smoothing, models 3 and 4, respectively) did not show a significant improvement on model fit compared to the fitted model with independent random effects (Table 1). Therefore, the model with independent random effects was selected as the final model. Sex, deprivation index, and urbanicity were the covariates that better explained case distribution in the region (Table 2). We explored adding Hillshade as a covariate, but it did not improve model fit and led to slightly decreased dispersion of the residuals and was therefore discarded. Figure 3 shows the fitted aSIR values throughout Gran Canaria with the final model (modeled aSIR with fixed effects—the explanatory covariates—and with independent random effects).

**Table 2:** Posterior median and 95% credible intervals for the fixed effects of the final model for the distribution of skin melanoma incidence in Gran Canaria, 2007–2018

Covariates	Regression coefficient	95% credible interval (CI)
Intercept	0.84	(0.77; 0.92)
Proportion of females	1.09	(1.00; 1.18)
Deprivation index	0.86	(0.79; 0.94)
Urbanicity	1.13	(1.03; 1.23)
Solar radiation	1.00	(0.92; 1.09)

Final model: Bayesian conditional autoregressive model with fixed effects and independent random effects

## Discussion

We found evidence of spatial heterogeneity in the distribution of skin melanoma incidence in Gran Canaria for the period 2007–2018, at the CT level (Moran’s *I* = 0.06, pseudo *p*-value = 0.009). Hot spots were identified in northern urban CTs, whereas a few cold spots clustered in central non-urban CTs (Figs. 1, 2). In the Bayesian multivariable model, a high SES and urbanicity were independently associated with an increased incidence (Table 2). This supports the existence of a social and an urban–rural gradient in melanoma incidence in the island and adds to the international body of evidence that high-SES populations present higher rates of skin melanoma [3].

Previous studies have suggested that high-SES populations might present higher rates of disease due to an easier access to recreational sun-related activities; hence, more sunburns and melanoma risk [3, 40, 41]. However, Gran Canaria is a sun-and-beach destination itself and the hypothesis of a differential accessibility to sun-related activities would not hold in this region. Additionally, low-SES CTs tended to suffer from higher solar radiation than high-SES ones (Supplementary Fig. S2), and lower-SES populations might struggle to buy sunscreen products. A differential sun-tanning behavior based on SES, with high-SES populations seeking sun tanning more intensively, rather than accessibility, could explain the social gradient in Gran Canaria [41].

Urbanicity was also associated with a higher incidence (Table 2). This is consistent with previous studies conducted in some Northern European countries and Canada [9, 38, 42]. Conversely, rurality was found to be a risk factor in Costa Rica [39], mostly linked to pesticide exposure. Urbanicity might also be associated with increased sun-seeking behaviors, with differential perceptions of tanned skin or clothing trends, posing individuals living in urban neighborhoods at an increased risk of melanoma [41]. Future qualitative studies in GC might help disentangle the mechanisms explaining these associations.

An alternative explanation of these social and urban–rural gradients might be the presence of a diagnostic access bias, with early-stage forms of melanoma going underdiagnosed in deprived and remote settings [3, 43]. Although Spain’s universal healthcare should make this hypothesis unlikely, a previous study showed that in northern Gran Canaria, a low educational attainment was associated with late-stage melanoma diagnoses [44]. Future research should aim at clarifying whether the aforementioned gradients reflect a health inequality [45].

The distribution of incidence was a revealing finding that might deserve further study. A radial pattern could be observed, overlapping to the ravines’ map of the island (Fig. 1 and supplementary Fig. S1). High values could be observed following the ravines of Tirajana, Telde, Azuaje, and end of Guiniguada. On the other hand, low values were found in the Arguineguín, Mogan, Tasartico, La Aldea, Moya, and Tenoya ravines. The regression model that showed better fitting of aSIR was a model with fixed effects (the selected explanatory covariates) and independent random effects. The latter, associated to each CT, are intended to account for any variance unexplained by the fixed effects and can be explained as unmeasured individual or CT factors which might have helped better explain the spatial distribution of skin melanoma (Table 1). Genetic and toxic factors are especially relevant in the epidemiology of geographically isolated areas, like islands [46], and might account for this uncaptured variability and explain this radial pattern. Historically, communication across the steep ravines was very difficult due to the lack of roads [47], favoring inbreeding and similar water supplies within ravines.

Distinct genetic populations might exist in Gran Canaria [48, 49] reflecting the influx of the varying ethnical groups that progressively populated the island. New mutations with founder effects have been discovered, with some genetic diseases clustering in particular ravines [50, 51]. Gran Canaria islanders are fundamentally of Spanish descent [52], due to the colonization process occurring in the fifteenth century by the Crown of Castile [49, 53, 54]. Settlers from Italy, Portugal, Flanders, and north African territories represented significant population influxes during the sixteenth century onward [55] and tended to group together according to origin [49, 55]. British communities followed later on, settling predominantly in urban regions of Gran Canaria capital city, LPGC [56–59]. Particularly, British merchants established in the commercial area of Triana and around the modern La Luz port [51, 57, 60] of LPGC, where a hotspot was identified (Fig. 2). Genetic susceptibility to melanoma is still poorly understood [61], and the particular distribution of cases in Gran Canaria, together with its anthropological evolution, might render the island a setting of particular interest for future genetic research.

An alternative explanation of the radial pattern could be related to water toxicity. Radon and pesticides have been attributed a potential causal role in skin melanoma [39, 62–64]. The cleanest waters of the island are considered to be in the top central areas of Tejeda and Artenara, where spring water is available. Those municipalities were classically the most isolated areas of the island, with the higher intensities of solar radiation, yet they constitute the predominant cold spot of incidence. Additionally, the Azuaje, Guiniguada, and Tirajana ravines (regions with high incidence) have long suffered from polluted waters [65–68]. A comparative study of water composition across ravines would allow to test potential associations between selected chemicals and skin melanoma occurrence. Future research should aim at clarifying whether genetic, toxic factors, or both might better explain the radial pattern of skin melanoma incidence in Gran Canaria.

Regarding the covariates used, this study supports the validity of IP2011 [19] as a measure to assess SES in Spain [69]. Despite SES being a multidimensional construct, most health studies have used a single variable to adjust for SES, either related with education, wealth, or occupation, but seldom integrating many of them, which might have led to biased adjustments [3, 70]. Deprivation indexes have been developed in some regions to overcome these limitations [71]. Until very recently, Spain had a deprivation index available for major cities [72] but not for the whole extension of the country. In this study, IP2011 correlated well with wealth and educational variables others than the ones used for its composition (Supplementary Fig. S3).

Our study has some limitations. First, the relatively low number of cases per year did not allow to evaluate the temporal fluctuations within the study period, nor to perform separate calculations for men and women. The effect of sex on incidence was therefore measured as percent of females per CT and the population denominator of the 2011 neighborhood considered as fixed for the entire study period, which might have produced residual confounding.

Second, our assessment of the role of solar radiation might not have provided an adequate measurement of the effect of this variable. Epidemiological studies using a small-area approach are ecologic, and exposure estimates of covariates are assigned to individuals based on location of residence. This approach has been validated for demographic and socio-economic variables [73], but might be more limited for solar radiation. Due to data availability, exposure was estimated at the place of residence, at the time of diagnosis. Yet a long spatial lag is expected between sun exposure and melanoma development [40, 74].

Finally, this study relies on the assumption that all incident skin melanoma cases diagnosed in Gran Canaria residents in the study period were considered. Because of how the dataset was elaborated, we have certainty that all cases diagnosed in the public healthcare system (CIHS) were included, but a few cases diagnosed in private practices might have been missed [13]. The CIHS covers about 87% of all healthcare in the region, and cases diagnosed in private clinics in our dataset represented 15.8%, that is, more than expected [13, 15]. Therefore, the plausible number of missing cases is unlikely to have meaningfully biased the results. Moreover, the uptake of private healthcare services in Spain is more common among high-SES individuals [75], and the plausible missing cases would be more likely to reside in high-SES neighborhoods, thereby increasing the effect estimates found.

Our study also has many strengths. The small-area approach used allowed the integration of individual-based health data and high-resolution small-area data, providing high-level granularity. This “semi-ecological” design is considered to allow a finer adjustment than traditional ecological studies, thereby diminishing the risk for ecological fallacy and improving causal inference [73].

The geospatial approach allowed disease mapping in Gran Canaria, providing the first identification of high- and low-risk areas in the region. It also establishes the role of demographic, socio-economic, and environmental variables in the relative risk of skin melanoma occurrence in Gran Canaria and provides new opportunities to assess their impact from a public health perspective [76]. Both the insularity and the anthropological evolution of Gran Canaria might provide a unique setting to test unresolved questions in melanoma epidemiology and pathogenesis.

## Conclusion

We found evidence of spatial heterogeneity in the distribution of skin melanoma incidence in Gran Canaria in 2007–2018 at the CT level. High values were identified in northern urban CTs and in certain ravines of the island, whereas a few cold spots clustered in central non-urban territories. A high SES and urbanicity were independently associated with increased incidence. Future studies will need to address whether SES and urbanicity truly explain differential melanoma susceptibility in the region, or whether they might be the result of health inequalities in skin melanoma detection. The clustering of high values following some ravines with historical isolation might represent an opportunity for future discoveries of the potential role of genetic and toxic factors in melanoma pathogenesis.

## Supplementary Information

Below is the link to the electronic supplementary material.

Supplementary file1 (DOCX 2699 kb)

## Data Availability

The data that support the findings of this study are available on reasonable request from the corresponding author (aggregated data). The data are not publicly available due to privacy or ethical restrictions.
