# Supplementary material for: Assessing the effect of environmental and socio-economic factors on skin melanoma incidence: an island-wide spatial study in Gran Canaria (Spain), 2007–2018
Source: Cancer Causes Control. 2022 Aug 4;33(10):1261–72. doi: 10.1007/s10552-022-01614-6 (PMC9427872; doi:10.1007/s10552-022-01614-6)
Supplement: Supplementary file 1 — Supplementary file1 (DOCX 2699 kb) [file 10552_2022_1614_MOESM1_ESM.docx]

**Supplementary File**

**Assessing the effect of environmental and socio-economic factors on skin melanoma incidence: an island-wide spatial study in Gran Canaria (Spain), 2007-2018**

**Table S1**. Description of covariates considered for modelling the incidence of invasive skin melanoma in Gran Canaria 2007-2018………………………………………………………………....2

***Table S2****. Univariable Poisson regression models of covariates potentially affecting the risk of skin melanoma in Gran Canaria (2007-2018), with ordinary least squares estimation………….4*

**Figure S1**: Ravines and Basins of Gran Canaria……………………………………………………...6

**Figure S2**: Quintile maps, distribution of potential covariates (Deprivation Index, Percent of females, percent of aged>64 years old, percent of urbanized land, Altitude SRTM250, Solar radiation, Hillshade)……………………………………………………………………………………….7

**Figure S3**: Correlogram of all covariates (demographic, socio-economic and environmental) included in the modelling. Pearson’s correlation test was used to explore the collinearity between pairs of covariates………………………………………………………………...………………………11

**Text T1.** Details on the implementation of Bayesian statistical modelling…………………………13

Table S1. Description of covariates considered for modelling the incidence of invasive skin melanoma in Gran Canaria 2007-2018

| **Covariate class** | **Description of covariate** | | **Time period** | **Source of data (format)** |
| --- | --- | --- | --- | --- |
| Demographic | Total population | | 2011 | Spanish Population Census of 2011 at the CT level, Spain’s National Institute of Statistics (shapefile format):  https://www.ine.es/en/censos2011_datos/cen11_datos_resultados_rejillas_en.htm |
|  | Number of males | |  |  |
|  | Number of females | |  |  |
|  | Persons aged under 16 years | |  |  |
|  | Persons aged between 16 (included) and 64 (included) years | |  |  |
|  | Persons aged over 64 years | |  |  |
| Socio-economic | Illiterate persons | “basic studies” |  |  |
|  | Persons without studies (<5 years of school attendance) |  |  |  |
|  | Persons with 1^st^ level studies (elementary school completed as higher educational attainment) |  |  |  |
|  | Persons with 2^nd^ level studies | |  |  |
|  | Persons with 3^rd^ level studies | |  |  |
|  | Persons without information regarding educational level (age <16 y) | |  |  |
|  | % of population with income per consumption unit (pcu) < 5000 euros/year | | 2015 | Spain’s Household Income Atlas - 2015 Economic indicators at the CT level, Spain’s National Institute of Statistics (shapefile format):  <https://www.ine.es/dynt3/inebase/en/index.htm?padre=5608&capsel=5650> |
|  | % of population with income pcu < 7500 euros/year | |  |  |
|  | % of population with income pcu <10000 euros/year | |  |  |
|  | % of population depending on salary | |  |  |
|  | % of population depending on unemployment subsidy | |  |  |
|  | % of population depending on retirement pension | |  |  |
|  | Deprivation index (IP2011): Standardised index with mean of 0 (=mean deprivation in Spain), standard deviation of 1. Positive values indicate higher deprivation. | | 2011 | Spanish Society of Epidemiology - At the CT level (shapefile format)  https://seepidemiologia.es/determinantes-sociales-de-la-salud/ |
| Environmental | Land cover data: Corine Land Cover (CLC) 2018. Raster at 100m resolution. | | 2017-2018 | European Environmental Agency, Copernicus Project (raster format)  https://land.copernicus.eu/pan-european/corine-land-cover/clc2018 |
|  | Reclassified landcover data obtained from CLC 2018: Classes grouped into major groups; artificial surfaces (Urbanized Land), forests, semi-natural areas, agricultural areas, and bare soil.  Raster further reclassified as % of urbanized land, and % of nonurban land. | | 2017-2018 | (Raster format) |
|  | Climatic data: Solar Radiation. Raster of solar radiation (in kJ m-2 day-1) | | 1970-2000 | Worldclim (raster format):  http://worldclim.org/data/worldclim21.html |
|  | Elevation data: Resampled Digital Elevation Model (DEM) data at 250m resolution. | | 2011 | (Raster format)  https://cgiarcsi.community/data/srtm-90m-digital-elevation-database-v4-1/ |
|  | Slope: From ArcGIS Slope function, based on DEM data (in degrees) | |  | https://pro.arcgis.com/en/pro-app/2.7/help/analysis/raster-functions/slope-function.htm |
|  | Hillshade: From ArcGIS hillshade function, based on azimuth and altitude. It’s a tridimensional representation of the surface considering the sun’s relative position | |  | https://desktop.arcgis.com/en/arcmap/10.3/manage-data/raster-and-images/hillshade-function.htm |
|  | Cartography | | 2011 | Spain’s National Institute of Statistics – At the CT level (shapefile format):  <https://www.ine.es/ss/Satellite?c=Page&p=1259952026632&pagename=ProductosYServicios%2FPYSLayout&cid=1259952026632&L=1> |

CT: Census Tract

***Table S2****. Univariable Poisson regression models of covariates potentially affecting the risk of skin melanoma in Gran Canaria (2007-2018), with ordinary least squares estimation*

| **Variable** | **Regression Coefficient (Standard error)** | **AIC** | **p-value** |
| --- | --- | --- | --- |
| Demographic and socio-economic factors | | | |
| **% of females** | 2.67 (1.0) | 1753.2 | 0.007 |
| **Deprivation index IP2011** | -0.20 (0.1) | 1745.0 | <0.001 |
| p_basic | -0.76 (0.4) | 1757.0 | 0.07 |
| p_2_grd | -0.83 (0.6) | 1758.2 | 0.14 |
| p_3_grd | 1.14 (0.4) | 1751.0 | 0.002 |
| R_prs15 | 0.00007 (0.00002) | 1742.4 | <0.001 |
| Rhog15 | 0.00003 (0.000006) | 1742.2 | <0.001 |
| ing_s_15 | -0.007 (0.05) | 1758.3 | 0.15 |
| ing_p_15 | 0.009 (0.007) | 1758.5 | 0.17 |
| ing_in_15 | -0.06 (0.02) | 1753.7 | 0.006 |
| ot_p_15 | -0.04 (0.03) | 1757.8 | 0.11 |
| otrs_15 | 0.03 (0.007) | 1742.7 | <0.001 |
| al_5_15 | -0.01 (0.00) | 1759.4 | 0.32 |
| a_7.5_15 | -0.01 (0.00) | 1755.5 | 0.03 |
| a_10_15 | -0.01 (0.00) | 1749.7 | 0.001 |
| Environmental factors | | | |
| **ASYear** | -0.0000001 (0.00000008) | 1758.2 | 0.14 |
| ASMnthl | -0.0000007 (0.0000007) | 1759.3 | 0.30 |
| HllShd1 | -0.001 (0.002) | 1760.0 | 0.56 |
| HllShd2 | -0.004 (0.002) | 1756.3 | 0.05 |
| Slope | 0.0000002 (0.00000007) | 1755.0 | 0.02 |
| Altitude (SRTM) | -0.0004 (0.0002) | 1755.5 | 0.03 |
| Cropland | -0.59 (0.2) | 1752.1 | 0.004 |
| Veg-Forest | -0.47 (0.2) | 1752.3 | 0.004 |
| Water | -1.52 (2.5) | 1760.0 | 0.54 |
| Bare | 1.66 (1.7) | 1759.4 | 0.33 |
| **Urbanized** | 0.47 (0.1) | 1745.6 | <0.001 |

*For all models, the outcome considered is the age-adjusted standardised incidence ratio (aSIR) of skin melanoma in the study period, and the underlying spatial unit is the census tract. AIC: Akaike Information Criteria.*

*In bold, variables finally selected for the multivariable models (see main text and supplementary Fig. S3).*

*% of females: proportion of females over the total population*

*p_basic:* Proportion of individuals with elementary school as higher educational attainment (or less, includes illiterate) over the total of population aged over 16 years

*p_2_grd:* Proportion of individuals with 2^nd^ level studies as higher educational attainment over the total of population aged over 16 years

*p_3_grd:* Proportion of individuals with 3^rd^ level studies over the total of population aged over 16 years

*Rprs_15:* Average rent per person in 2015

*Rhog15:* Average rent per household in 2015

*ing_s_15:* % of population depending on salary in 2015

*ing_p_15:* % of population depending on retirement pension

*ing_in_2015:* % of population depending on unemployment subsidy in 2015

*ot_p_15:* % of population depending on another type of subsidy

*otrs_15:* % of population with different source of income

*al_5_15:* % of population with income per consumption unit (pcu) < 5000 euros/year in 2015

*a_75_15:* % of population with income pcu < 7500 euros/year in 2015

*a_10_15:* % of population with income per consumption unit (pcu) <10000 euros/year in 2015

*ASYear:* Solar Radiation, yearly average

*ASMnthl:* Solar Radiation, monthly average

*HllShd1:* Hillshade with standard ArcGIS function

*HllShd2:* Hillshade assuming and azimuth and altitude

*Cropland:* % of cropland surface (agricultural areas) over the total cover land surface

*Veg_Forest:* % of forests and vegetation surface over the total land cover surface

*Water:* % of water surface over the total land cover surface

*Bare:* % of bare surface over the total land cover surface

*Urbanzd:* % of urbanized / artificial surface over the total land cover surface

**Figure S1**: Ravines and Basins of Gran Canaria


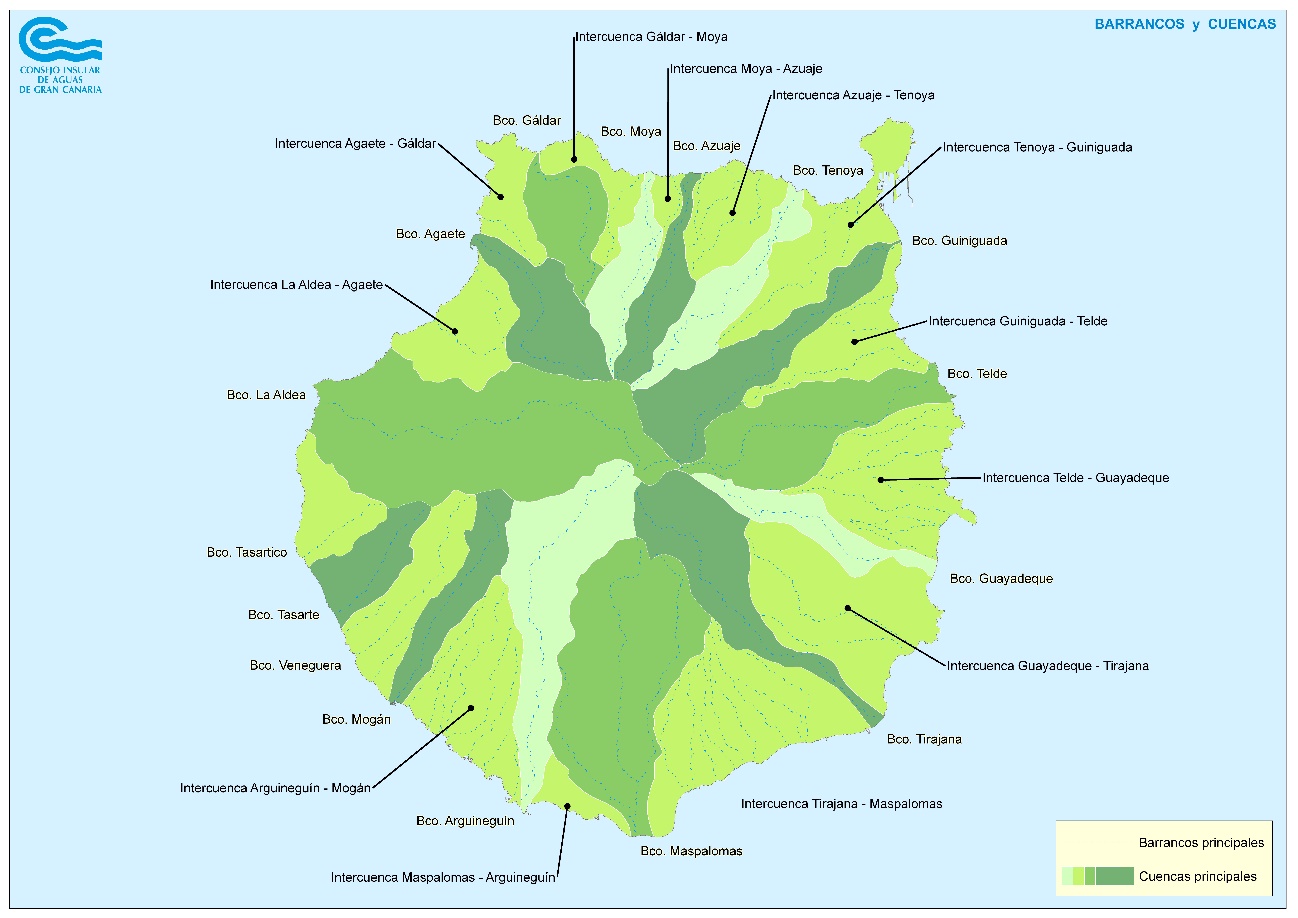


*Ravines indicated by the word “Barrancos” and the abbreviation “Bco.”. Basins indicated by the word “Cuencas”.*

Reproduced with permission from the website of the Gran Canaria Island Water Council (Consejo Insular de Aguas de Gran Canaria). Available at: <http://www.aguasgrancanaria.com/cartografia/medio_fisico/barrancos_cuencas.php>

**Figure S2**: Quintile maps, distribution of potential covariates (Deprivation Index, Percent of females, percent of aged>64 years old, percent of urbanized land, Altitude SRTM250, Solar radiation, Hillshade)

Gran Canaria City: LPCG


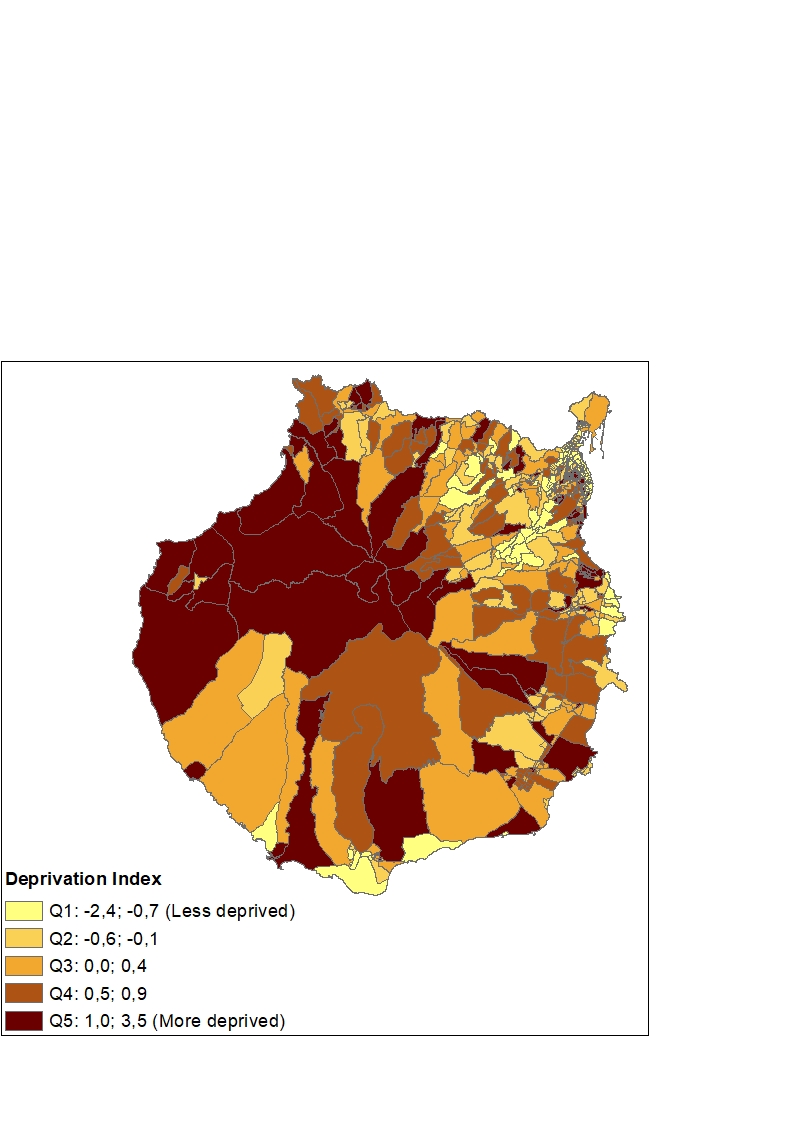

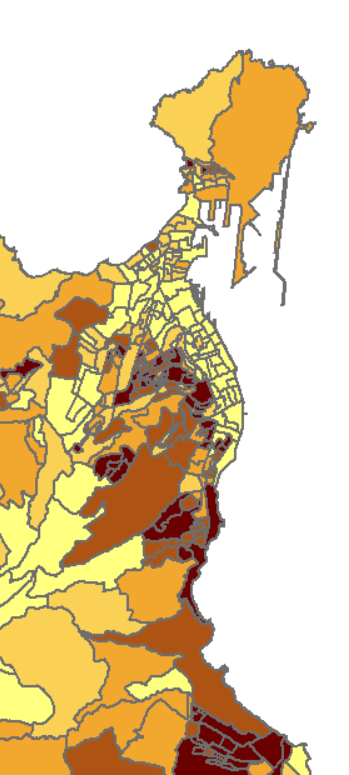


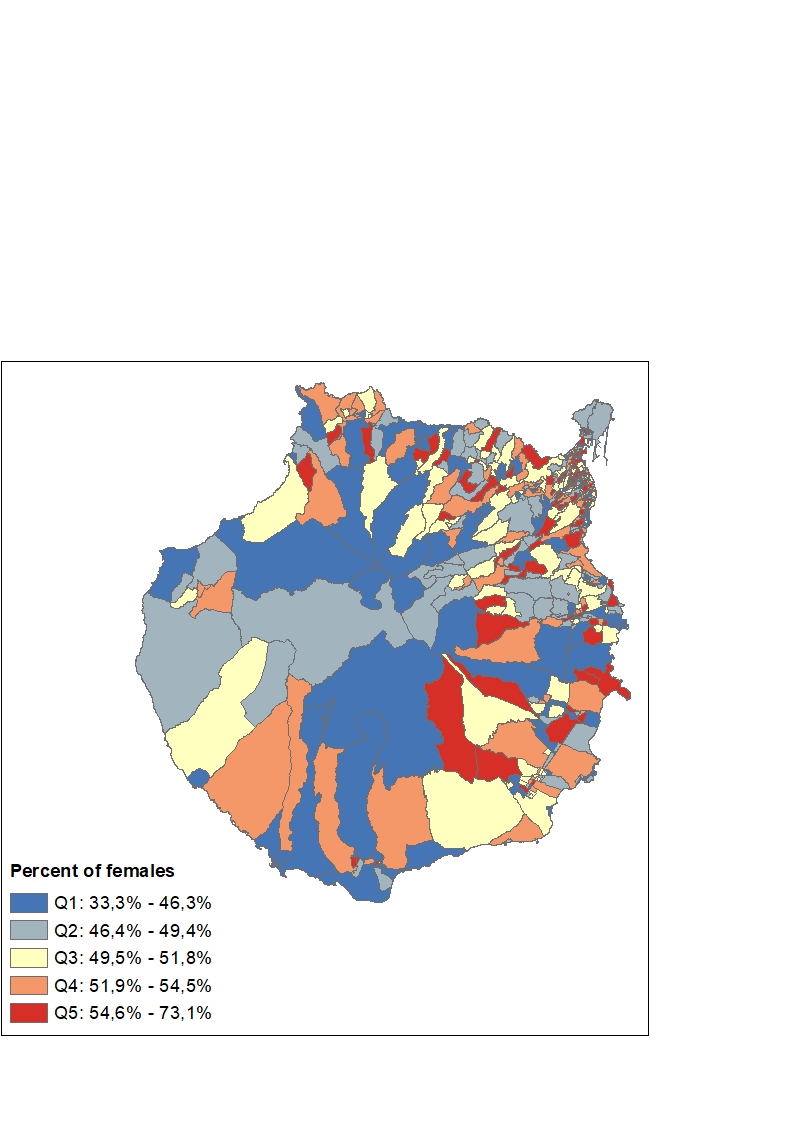

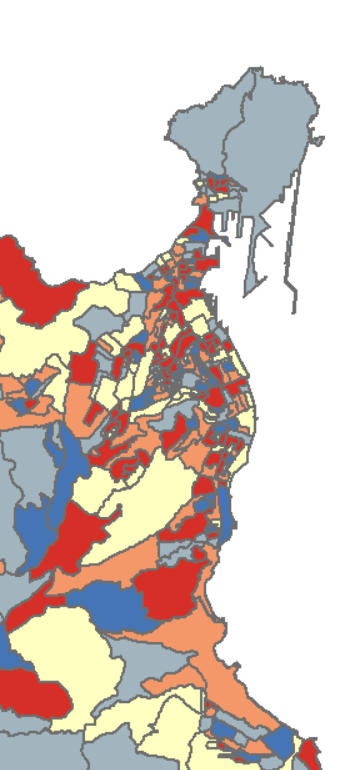


Gran Canaria City: LPCG


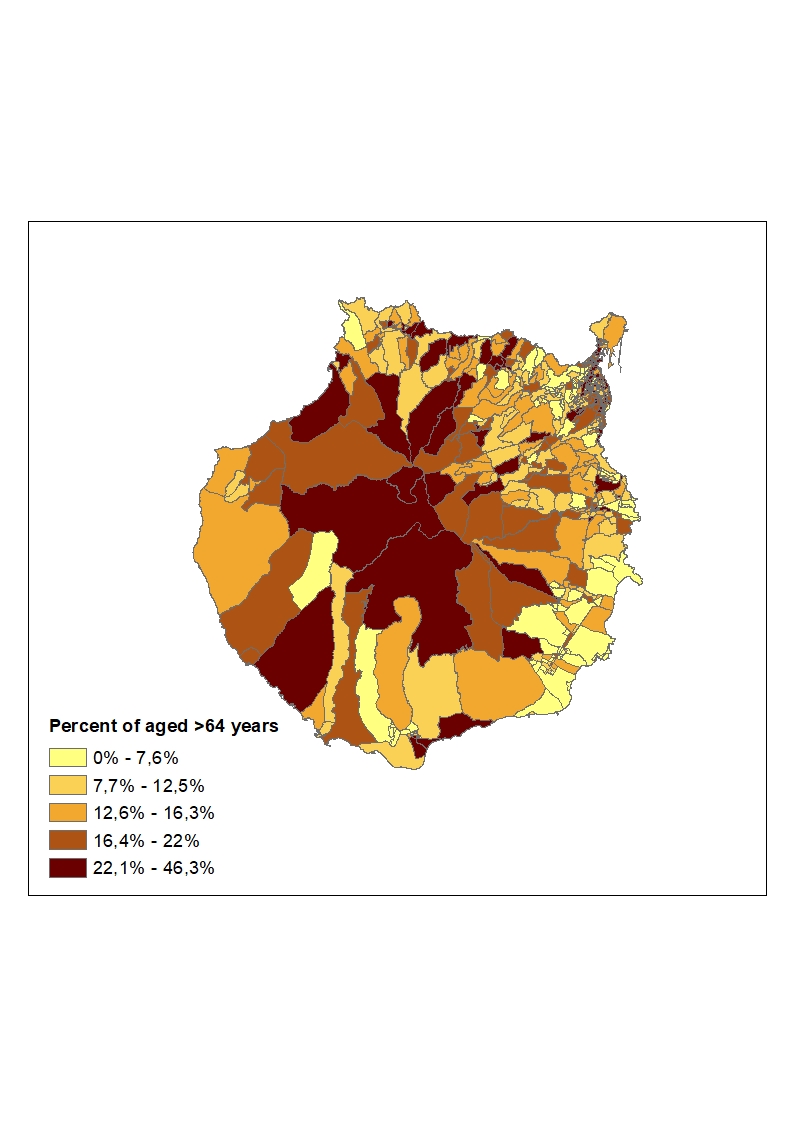

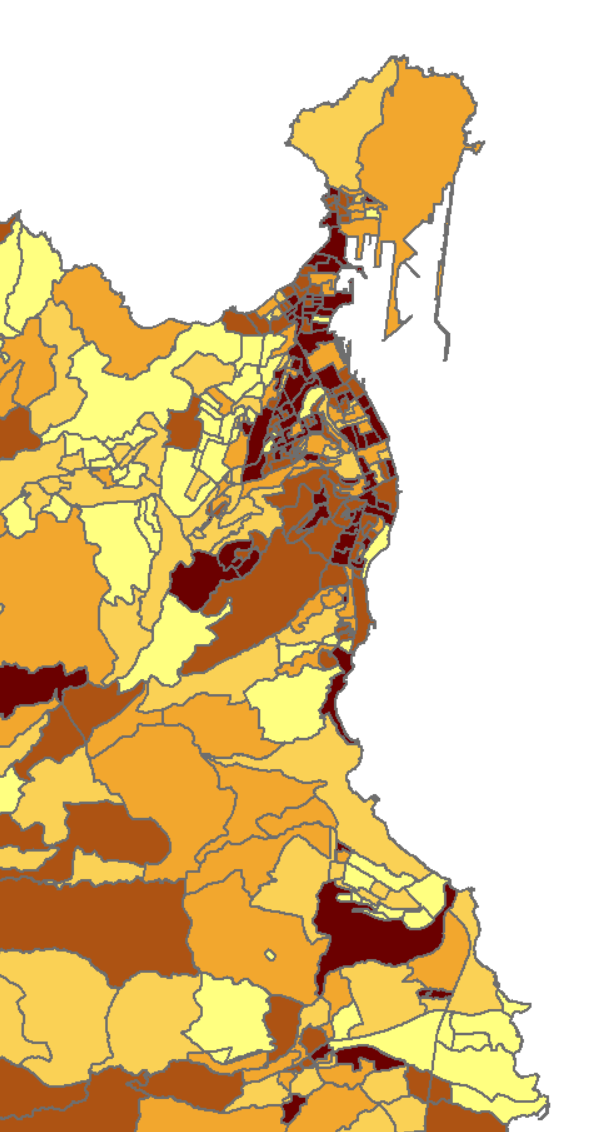


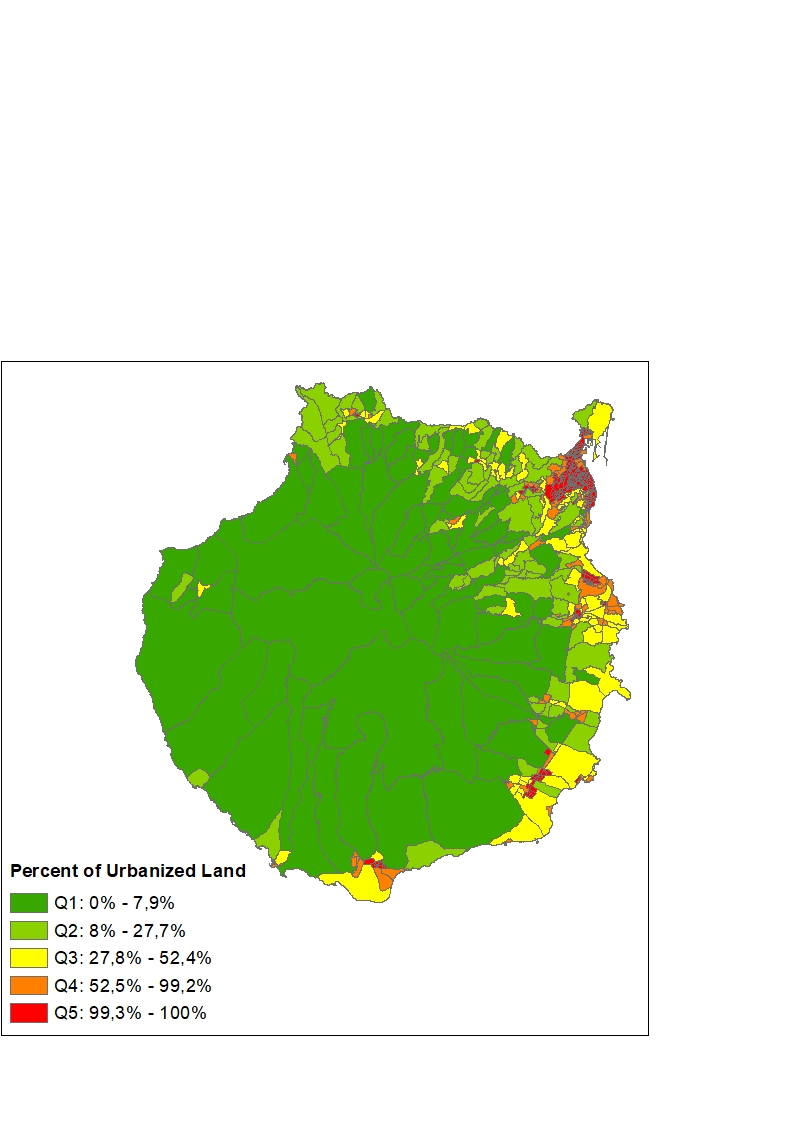

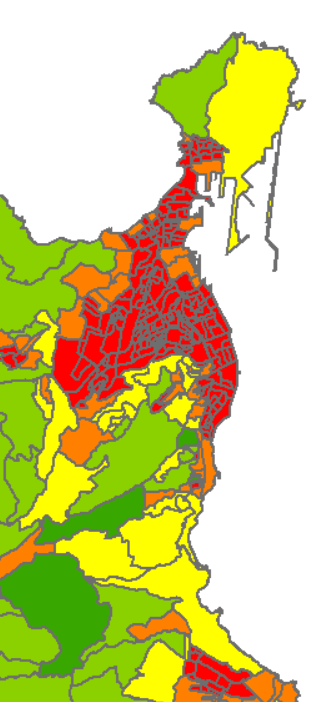


Gran Canaria City: LPCG


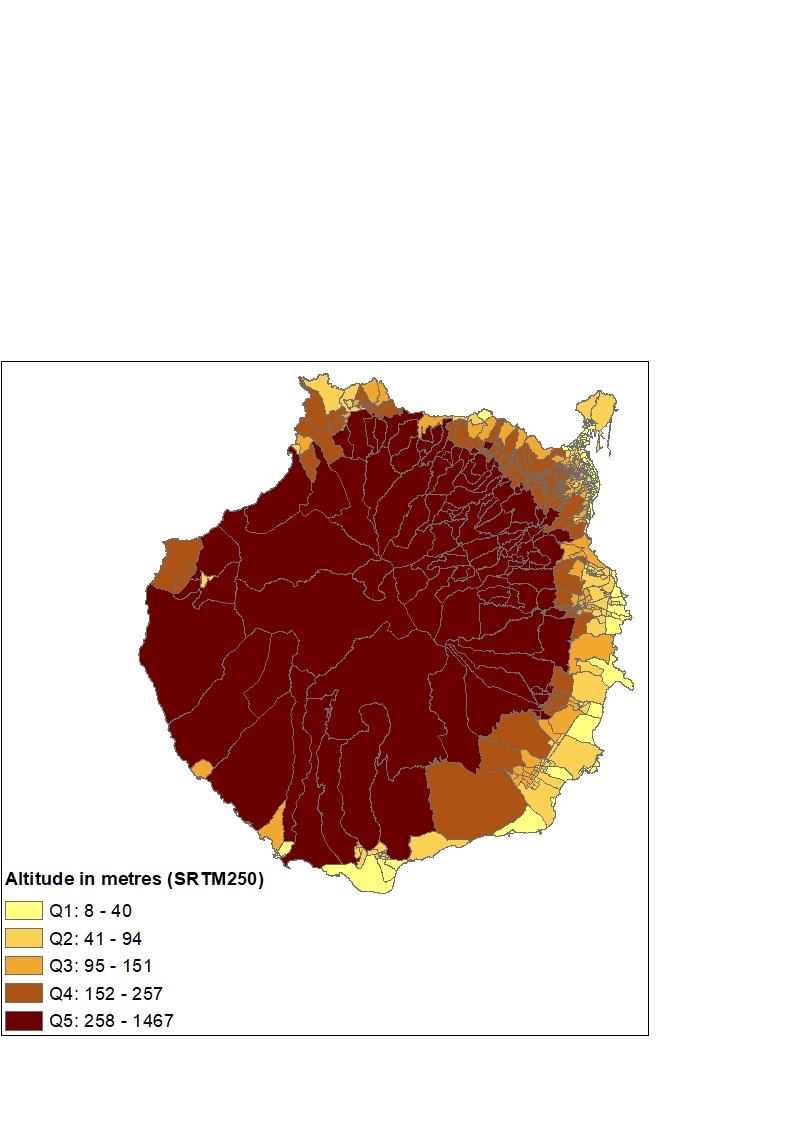

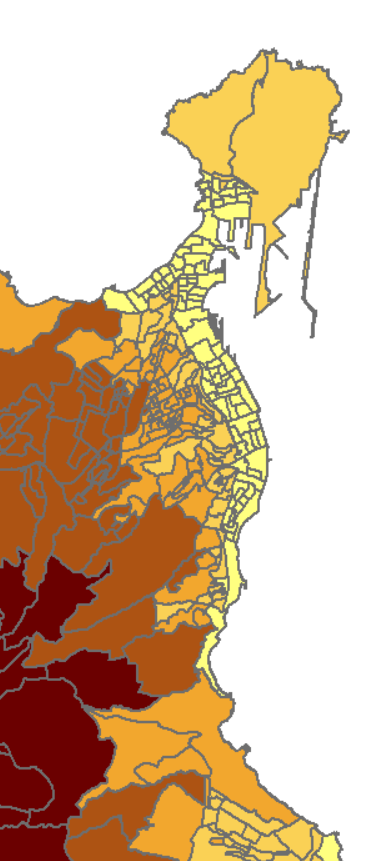


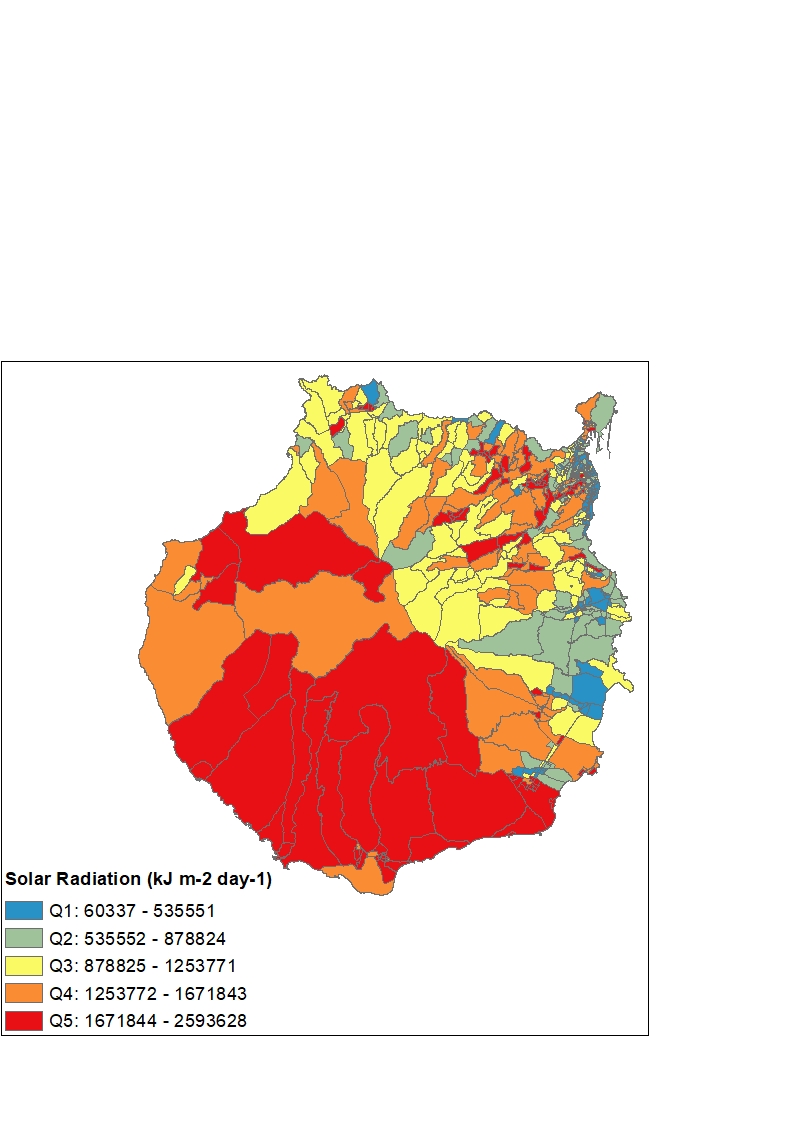

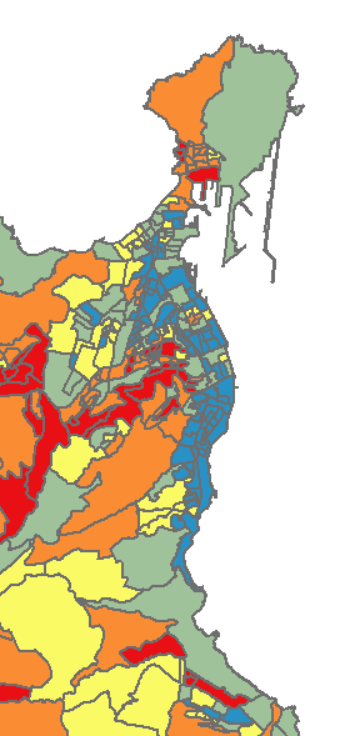


Gran Canaria City: LPCG


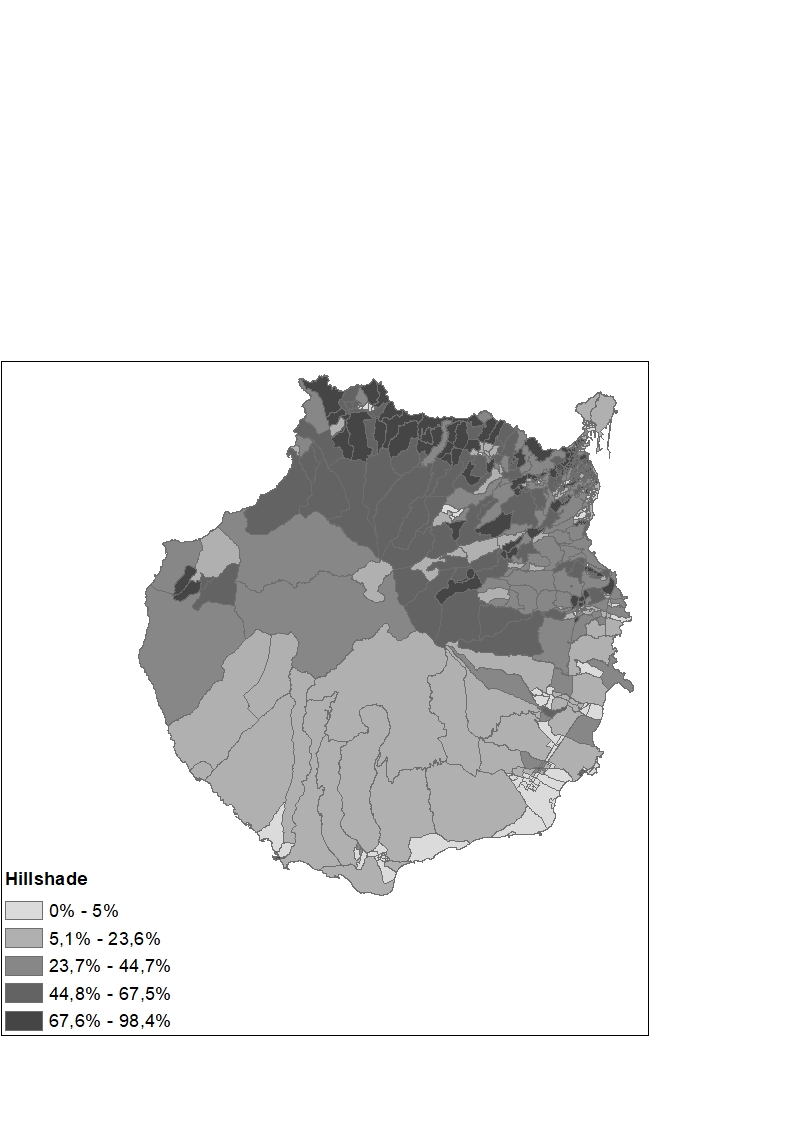

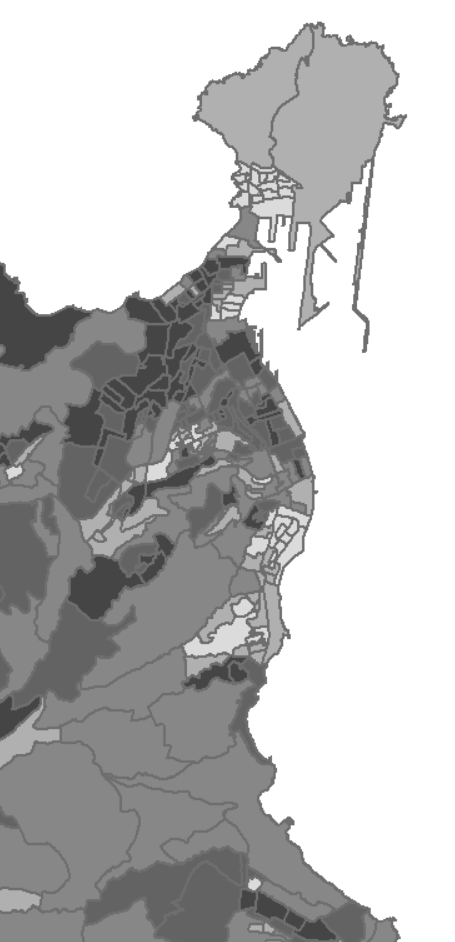


**Figure S3**: Correlogram of all covariates (demographic, socio-economic and environmental) included in the modelling. Pearson’s correlation test was used to explore the collinearity between pairs of covariates.

*
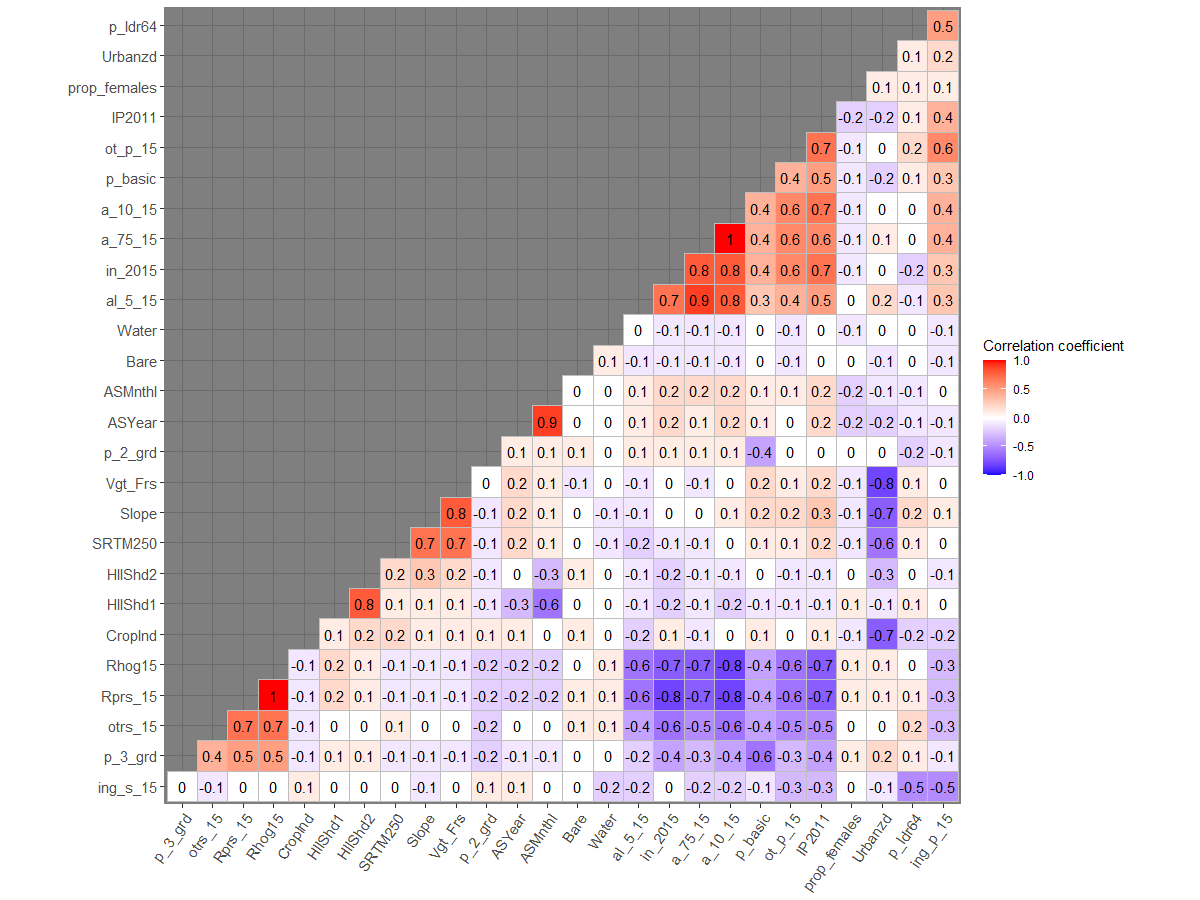
p_ldr64*: Proportion of people aged over 64, over the total population

*Urbanzd*: % of urbanized / artificial surface over the total land cover surface

Prop_*females*: proportion of females over the total population

*IP2011*: Deprivation index

*ot_p_15*: % of population depending on another type of subsidy

*p_basic*: Proportion of individuals with elementary school completed as higher educational attainment (or less, includes illiterate) over the total of population aged over 16 years.

*a_10_15*: % of population with income per consumption unit (pcu) <10000 euros/year in 2015

*a_75_15*: % of population with income pcu < 7500 euros/year in 2015

*in_2015*: % of population depending on unemployment subsidy in 2015

*al_5_15*: % of population with income per pcu < 5000 euros/year in 2015

*Water*: % of water surface over the total land cover surface

*Bare*: % of bare surface over the total land cover surface

*ASMnthl*: Solar Radiation, monthly average

*ASYear*: Solar Radiation, yearly average

*p_2_grd*: Proportion of individuals with 2^nd^ level studies as higher educational attainment over the total of population aged over 16 years

*Vgt_Frs*: % of forests and vegetation surface over the total land cover surface

*SRTM250*: Altitude

*HllShd2*: Hillshade assuming and azimuth and altitude

*HllShd1*: Hillshade with standard ArcGIS function

*Croplnd*: % of cropland surface (agricultural areas) over the total cover land surface

*Rhog15*: Average rent per household in 2015

*Rprs_15*: Average rent per person in 2015

*otrs_15*: % of population with different source of income

*p_3_grd*: Proportion of individuals with 3rd level studies over the total of population aged over 16 years.

*ing_s_15*: % of population depending on salary in 2015

*ing_p_15*: % of population depending on retirement pension

**Text T1.** Details on the implementation of Bayesian statistical models

The skin melanoma incidence data was fitted as age-adjusted standardised incidence ratios (aSIR) using multiple Bayesian Poisson models, based on a selection of explanatory covariates as potential risk factors and different options for random effects with i) no random effects, ii) independent random effects, and iii) spatially correlated random effects, implemented through a conditional autoregressive model (CAR).

Let us first introduce the general formulation

$$\begin{matrix} Y_{i}|\mu_{i} & \sim& Poissson\left( \mu_{i} \right)i=1,\ldots,N \\ log\left( \mu_{i} \right) & = & x_{i}^{T}\beta+O_{i}+U_{i} \\ \beta& \sim& N\left( \mu_{\beta},\Sigma_{\beta} \right) \end{matrix}$$

where $x_{i}$ was a $\left( p+1 \right)\times1$ vector of known exploratory covariates that were suspected to be risk factors for the disease, $O_{i}$ were known offsets, in our case the expected number of cases, and $U_{i}$ were random effects with a spatial structure to model the residual spatial variation that was not captured by the covariates.

1. The model without random effects. For this model, we initially fitted a Bayesian Poisson model with no random effects to the dataset. This corresponded to a model where $U_{i}=0$ for each observation.
2. The model with independent random effects. To capture the extra Poisson variability present in the data, we introduced a set of independent and normally distributed random effects. This equated to define $U_{i}\sim N\left( 0,\tau^{2} \right)$ in the general model previously introduced.
3. The globally smooth CAR model with spatially correlated random effects. For this model, we used the Besag-York-Mollie (BYM) model as described in Besag et al. in 1991 [1]. Here, two sets of random effects (independent and spatially structured) were fitted as follows:

$$U_{i}=\phi_{i}+\theta_{i}$$

$$\theta_{i}\sim N\left( 0, \sigma^{2} \right)$$

$$\phi_{i}\mid\phi_{-i}\sim N\left( \frac{\sum_{i=1}^{n} w_{ik}\phi_{i}}{\sum_{i=1}^{n} w_{ik}}, \frac{1}{\tau^{2}\sum_{i=1}^{n} w_{ik}} \right)$$

$$\log\sigma,\log\tau\sim\log Gamma(1, 0.0005)$$

Weekly informative priors were specified on the log of the independent random effect precision value 1/$\sigma$ and on the log of the spatially structured effect precision $\tau$. Moreover, $w_{ik}=0$ was obtained if the census tract $i$ and $k$ did not share a border nor a corner and it they did share one or another $w_{ik}=1$ was obtained. These values were fixed and calculated *a priori* based on the adjacency of census tracts.

1. The locally smooth CAR model with spatially correlated random effects. The implemented model was described by Lee and Mitchell [2]. The random effects of the general model now had the following structure

$$\begin{matrix} U_{i}|U_{-i} & \sim& N\left( \frac{\rho\sum_{i=1}^{n} w_{ik}U_{i}}{\rho\sum_{i=1}^{n} w_{ik}+1-\rho},\frac{1}{\tau\left( \rho\sum_{i=1}^{n} w_{ik}+1-\rho\right)} \right) \\ logit\left( \rho\right) & \sim& N\left( 0,100 \right) \end{matrix}$$

$$\tau\sim Gamma(0.001, 0.001)$$

where, $\rho$ determined the global spatial correlation ($\rho=0$ corresponded to independence everywhere, whereas $\rho=1$ defined strong spatial correlation throughout the study region). Diffuse priors were assigned to both the precision parameter $\tau$ (Gamma) and the logit of $\rho$(Gaussian). The CT matrix elements $w$ were usually fixed as for the BYM model previously specified.

**REFERENCES**

1. Besag J, York J, Mollie A. Bayesian image restoration, with two applications in spatial statistics. Ann Inst Stat Math. 1991;43:1–20.

2. Lee D, Mitchell R. Locally adaptive spatial smoothing using conditional auto‐regressive models. J R Stat Soc Ser C (Appl Stat). 2013;62:593–608.
